# Systemic Racism in Canadian Occupational Therapy: A Qualitative Study with Therapists

**DOI:** 10.1177/00084174211066676

**Published:** 2022-01-05

**Authors:** Brenda L. Beagan, Kaitlin R. Sibbald, Stephanie R. Bizzeth, Tara M. Pride

**Keywords:** Minority groups, Power, Power inequities, Qualitative research, Groupes minoritaires, ‌inégalité de pouvoir, pouvoir, recherche qualitative

## Abstract

**Background.** Research on racism within occupational therapy is scant, though there are hints that racialized therapists struggle. **Purpose.** This paper examines experiences of racism in occupational therapy, including coping strategies and resistance. **Method.** Ten therapists from racialized groups (not including Indigenous peoples) were recruited for cross-Canada, in-person or telephone interviews. Transcripts were coded and inductively analysed, with data thematically organized by types of racism and responses. **Findings.** Interpersonal racism involving clients, students, colleagues and managers is supported by institutional racism when incidents of racism are met with inaction, and racialized therapists are rarely in leadership roles. Structural racism means the experiences of racialized people are negated within the profession. Cognitive sense-making becomes a key coping strategy, especially when resistance is costly. **Implications.** Peer supports and community building among racialized therapists may be beneficial, but dismantling structures of racism demands interrogating how whiteness is built into business-as-usual in occupational therapy.

## Résumé

**Description.** Les recherches sur le racisme dans le domaine de l’ergothérapie sont rares, mais des indices laissent entrevoir les difficultés qu’éprouvent les thérapeutes racisés. **But.** Le présent article examine les expériences de racisme dans le domaine de l’ergothérapie, ainsi que les stratégies d’adaptation et de résistance. **Méthodologie.** Dix ergothérapeutes issus de groupes racisés au Canada (excluant les Autochtones) ont été recrutés pour des entrevues téléphoniques ou en personne. Les transcriptions ont été codées et ont fait l’objet d’une analyse inductive, avec les données organisées thématiquement par type de racisme et type de réponse. **Résultats.** Le racisme interpersonnel associé à des clients, à des étudiants, à des collègues et à des gestionnaires est soutenu par un racisme institutionnel lorsque les incidents de racisme ne sont pas traités de manière proactive, et les ergothérapeutes racisés occupent rarement des postes de direction. Le racisme structurel fait en sorte que les expériences des personnes racisées sont niées au sein de la profession. Les stratégies d’adaptation cognitives jouent alors un rôle clé, surtout lorsque la résistance est coûteuse. **Conséquences.** Le soutien des pairs et l’établissement de communautés entre thérapeutes racisés peut être bénéfique, mais le démantèlement des structures racistes nécessite une remise en question de la manière dont la blanchité fait partie intégrante de l’ergothérapie.

## Introduction

Occupational therapy lacks research on racism within the profession. A system of oppression that permeates Western societies, racism is foundational to the colonial capitalist sociopolitical economy of Canada. In a nation constructed through colonial race relations, it would be surprising to find any institution—in fact, any sphere of social life—*not* affected by pervasive racism. Yet it remains important to critically analyse racism within specific social contexts, to better understand the processes through which forms of racism are integrated into routine everyday practice. Such critical analysis can help to identify effective avenues for change. This paper examines the professional experiences of 10 racialized occupational therapists in Canada, examining how structural racism infuses institutional and interpersonal practices, and how coping and resistance strategies are themselves complicated by racism. It does not include the experiences of Indigenous therapists, which will be addressed in future analyses.

## Background

### Conceptualizing Racism

“Race” is not a biologically meaningful category, but nonetheless holds very real social consequences, due to racism. Racism is a system of social power relations rooted in history and operating at multiple levels, through myriad intersecting social, political, and economic avenues. “Racialized” groups are those marked as subordinate in the sociopolitical and historical process of categorizing groups hierarchically (Miles, 1989). In other words, “race” is constructed as real, and as mattering, through racism. There are different ways of categorizing types of racism; here we employ the framework of interpersonal, institutional, and structural racism explicated by Nazroo et al. (2019). They emphasize the interconnections across levels that construct the all-encompassing nature of racism.

*Interpersonal* racism includes the everyday slights, prejudices, and discrimination occurring in routine interactions that range from looks, jokes, and comments, to actual physical violence (Essed, 1991). It includes what is often termed “microaggressions” in psychology (Sue, 2010), ostensibly minor incidents that add to an exhausting cumulative burden. Interpersonal racism can be experienced directly as well as vicariously, as the targeting of someone else from the same racialized group may feel personally threatening or painful (Essed, 1991). Racism, like all forms of oppression, targets people not as individuals but as members of their social group (Frye, 1983). Interpersonal racism is always an instantiation of broader structural racism—that is the source of its power. At the same time, interpersonal racism enacts, shapes, and reshapes institutional and structural racism. In other words, racism played out through institutions inevitably involves persons.

For Nazroo et al. (2019), *institutional* racism involves systems of organizational operations that arise from and reproduce racism through routine practices, policies, and procedures. Standards, rules, operating processes, and expectations—such institutional features are ostensibly race-neutral, yet operate to systematically advantage white, dominant group members, while excluding or disadvantaging racialized people. Institutions exist at the nexus of structural and interpersonal racism: though institutional practices are implemented by individuals, interpersonally, they also comprise the concentration or sedimentation of structural racism, through disembodied practices that are challenging to change (Nazroo et al., 2019).

*Structural* racism in this framework refers to the patterned social arrangements that result in racialized group members having inequitable access to and advantage through material, economic and sociopolitical resources (Nazroo et al., 2019). It incorporates what is often called cultural or ideological racism, ideas and beliefs that justify oppression. Ideas as commonplace as a meritocracy, and what counts as valued or authoritative or “normal,” are cornerstones of structural racism. Such ideas often come to hold their valued place in institutions because of the systematic erasure and silencing of alternative knowledge and values, while establishing white ways of knowing as legitimate and authoritative (Dotson, 2011; Mills, 2007). As is well-established in critical race theory, there is no “neutral” institution or social structure, which may or may not be “tainted” by aberrant racism; rather, white supremacy and systemic racism are normative, shaping the *status quo* of institutions and social arrangements in ways that privilege some and harm others (Crenshaw et al., 1995).

Some theoretical frameworks include “internalized racism” as a distinct form of racism (Vazir et al., 2019). We see this, rather, as one of the psycho-emotional harms of racism, in particular, a form of ontological harm, a harm “against being or existence” (Goodley & Runswick-Cole, 2011, p. 607), undermining one’s very right to exist and invalidating one’s sense of intrinsic value. Self-hatred, doubt, and low self-esteem—these are not signs of (pathological) “internalized racism,” they are products of systemic racism experienced at structural, institutional, and interpersonal levels. People bearing the brunt of racism also develop tremendous strengths and complex survival skills (Gibson, 2020).

### Racism in the Health Professions and Occupational Therapy

Systemic racism is evident in the health professions. In nursing, the culture of white dominance results not only in the lack of racialized nurses in leadership roles (Iheduru-Anderson & Wahi, 2021; Premji & Etowa, 2014), but also in racialized nurses experiencing hostility and microaggressions from both patients and coworkers (Cottingham et al., 2018). The only study of racism in Canadian physiotherapy highlighted the whiteness of the profession (Vazir et al., 2019). Racialized physiotherapists identified the culture of their profession, from assessment tools to who is in management, as a culture of whiteness into which they were expected to assimilate.

Despite the fact that occupation is inherently racialized, both structured by and structuring racism (Lavalley & Johnson, 2020), there has been little exploration of racism in occupational therapy. Ramugondo (2018) has argued that racism and colonialism, along with neoliberal capitalism, are foundational to the entire global economy, rife as it is with inequalities. Hammell (2019) notes that the imposition of theories and models of practice from the global North on occupational therapy in the global South is yet another instance of such neoliberal colonial racism. Indigenous scholars such as Emery-Whittington (2021) and Gibson (2020) have argued compellingly that decolonizing occupational therapy requires decentering whiteness. In Canada, Grenier (2020) has documented how white supremacy is implicated in the foundations of occupational therapy, defining white supremacy as not only the presumed superiority of white people, but also the establishment of white people and their/our ways as the standard for being human. She notes how everything from assessment norms to orthosis materials assume whiteness, how values such as occupational balance and categories of occupational domains are steeped in white Western culture. Even relatively new concepts such as occupational justice have been shown to be infused with the power relations of colonial racism (Emery-Whittington, 2021).

There is almost no evidence regarding the experiences of racialized occupational therapists. In Ireland, a qualitative study found occupational therapists who identified as ethnoracial minorities experienced considerable professional marginalization (Beagan & Chacala, 2012), reporting that core values of the profession were a poor fit with their own. They struggled with social aspects of workplaces, feeling like outsiders, and faced both microaggressions and overt racism from colleagues and clients. Racism from clients was particularly challenging to address, given the primacy of client-centred practice. One therapist who confronted a client's overt racism concluded, “I did do the wrong thing… It's the client's wish that is paramount. What I was doing was putting my wish to be accepted as a person, to be recognised as a person” (p. 149). This was the only incident recounted wherein the therapist confronted a client, and in no instance did participants bring racism to the attention of their managers, “lest they appear incompetent and put their jobs at risk” (p. 149). That study concluded there is little point in recruiting a diverse workforce in occupational therapy if no efforts are made to challenge exclusion and oppression within the profession. Perhaps unsurprisingly, an earlier UK survey of occupational therapists concluded that racialized therapists experienced barriers to career progression (Bogg et al., 2006).

In Canada, there have been calls since 2007 for research regarding the experiences of racialized occupational therapists (CAOT, 2007), yet there remains “a resounding silence on the issue of racism and its relationship to occupational therapy” (Beagan & Etowa, 2009, p. 285). Canada does not collect data on race within the profession, but calculating from the latest census data, “visible minority” therapists make up 13.7% of occupational therapists compared with 22.3% of the Canadian population (Statistics Canada, 2016). Understanding therapist experiences of racism—at interpersonal, institutional, and structural levels—is a step towards better understanding how whiteness infuses the profession, shaping expectations, assumptions, and perceptions of “normal” in ways that consistently privilege white occupants of the therapist role, reproducing racialized hierarchies. Social change demands accurate analysis, understanding of how racism operates to disrupt it.

With a critical race emphasis on counter-storytelling to surface subordinated realities (Crenshaw et al., 1995), this study asked: how do racialized occupational therapists in Canada experience racism within the profession, and what do they suggest regarding change? We recognize that this is not “news” to racialized therapists, yet trust that naming that reality can contribute to an epistemic disruption within a profession that prides itself on its work towards equity.

## Methods

We draw on a subset of data from a larger study examining the experiences of health professionals (physicians, nurses, and occupational therapists) who self-identify as disabled, working-class origin, racialized, ethnic minority, and/or minority sexual/gender identity. The experiences of Indigenous professionals will be a second phase of the research. The study was approved by three university research ethics boards. Grounded in critical theory, in-depth interviews were conducted with 49 participants from across Canada, exploring belonging and marginality, the toll of oppression, as well as coping and resistance. With consent, interviews were conducted by three researchers, all of whom identify as members of the groups being recruited. Interviews were conducted by phone or in-person, were recorded and transcribed, then coded using ATLAS.ti software.

This analysis included 10 occupational therapists, those who self-identified as racialized (see Table 1). Iterative analysis moved between compiling coded data (quotations) and re-reading full transcripts, with some codes emerging from the data (e.g., belonging and coping strategies), others from theories of racism (e.g., microaggressions and overt hostility). Repeated analysis and team discussion identified the conceptual framework above as best-suited to this part of our data, allowing us to examine how it could characterize participant narratives. This was not *a priori* coding based on a predetermined theory, rather it was immersion in the data, then sifting through theories of racism to best explain these data. Quotations were organized and reorganized as subthemes emerged, then “cleaned” by removing false starts and filler words such as “um” and “ah.” We chose not to use pseudonyms to avoid the violation of misnaming.

**Table 1.** Demographics.

ID#	Identities	Practice area/type		Rural/urban	Age, years in practice	Years in practice
OT2	East Asian	Continuing care		Rural and small city	35–45	10–15
OT4	African Canadian	Community		Small city	45–50	20–25
OT5	Black	Mental health		Large city	30–35	10–15
OT6	African Canadian	Mental health		Small city	40–45	20–25
OT13	West Asian	Mental Health		Large city	40–45	15–20
OT15	Southeast Asian	Community		Large city	30–35	5–10
OT16	West Asian	Community		Large city	30–35	5–10
OT17	East Asian	Private practice, community		Large city	30–35	5–10
OT18	East Asian	Community, insurance		Large city	30–35	5–10
OT19	Latin American	Community, hospital		Large city	30–35	5–10

### Limitations and Strategies for Rigour

While we report demographics, we deliberately keep details vague to reduce identifiability—which inevitably glosses over important differences. We did not employ member-checking, as previous experience has shown professionals rarely respond to preliminary analyses. The study is limited by having a relatively small sample that is also heterogeneous, including members of several racialized groups; this runs the risk of collapsing diverse experiences and losing identify-specific experiences. Recruitment was discontinued when the COVID-19 pandemic overwhelmed health professionals. Nonetheless, saturation was deemed to have been reached on most themes. The strength of a heterogeneous sample is that it allows exploration of racism experiences across multiple groups.

The members of the larger team encompass all the social groups included in the study, thus lived experience of oppressions in professional contexts informed every aspect of the study, from its conceptualization to analysis. Regular meetings of the team enabled reflexive analysis, challenging and building on each other’s interpretations. The author group for this paper includes occupational therapists and an outsider to the profession; it includes racialized and indigenous scholars, as well as White settler scholars who experience race-privilege. “Thinking together” allowed a kind of transpersonal reflexivity, making biases and preconceptions explicit rather than attempting to bracket them out (Dörfler & Stierand, 2021).

## Findings

Participants all identified as women, and included East Asian, Southeast Asian, African Canadian, and Latin American therapists (Table 1). All worked in cities (small and large), most in community contexts. Though some participants, especially first- and second-generation Canadians, thought their racialized and ethnic identities helped them connect with clients, those advantages were heavily outweighed by the negative experiences, challenges, and barriers therapists faced in their daily work. Below we explore how their professional experiences were shaped by interpersonal, institutional, and structural racism, and how coping and resistance strategies were themselves complicated by racism.

## Interpersonal Racism

Instances of interpersonal racism occurred with clients, students, colleagues, and managers, both directly and vicariously. Some instances could be considered “veiled racism,” racism masquerading as something else. Social exclusion was described by most participants: “Do I feel I fit in? Probably, to be honest with you, most of the time I don't… Even sometimes, in conversations.” Work-related social events were particularly challenging when groups of coworkers got together and the participant was not invited.

Some clients overtly refused to work with racialized therapists; all three African heritage therapists had had clients refuse to work with them because they are Black. Some refusals to work with racialized therapists employed “coded” language about accent or experience level:

[One client] told me that I had an accent and she couldn't understand me. Clearly, I don't have an accent. And it was clear that she just didn't like me because, for whatever reason. I can only attribute it to the fact that I am visibly Asian, because of the comments that she was making… It's happened more than once… That sort of situation, no matter what I’m saying, they’re just seeing somebody that they don't like. And they’re making comments about an accent that doesn't exist… They just don't like you, based on what you look like.

A participant working in academia found students attacked her religion, which was intertwined with her race and ethnicity:

I’ve definitely had some backlash… comments that I don't think my peers have ever received. I think it's a number of things there, around my age, my gender, and my identity in terms of race and also in terms of religious views. Anyway, the feedback that I got from students wasn't constructive. It was just very hurtful.

### Veiled Racism

This kind of “veiled racism” was also apparent in comments about ethnic and geographic origins. Asking “where are you from?” can be innocent, but can also convey messages of not-really-belonging here, messages of exclusion: “If they’re asking me where I’m from, because they know I’m not Canadian and I’m making mistakes when I’m talking, yeah, that's a little disrespectful.” From colleagues as well as clients, therapists faced relentless questions about their countries of origin (where they “really” belong) and when they were going “back home.” Frequent corrections to accent or spoken English seemed intended to humiliate; one participant had a boss correct her English in a meeting, in front of dozens of colleagues, which—as the only racialized person in the room—she experienced as bullying.

Sometimes participants were treated as exceptions from the general negative perception of their racialized group: “You are different from *those* people… I know you are Black, but when I talk to you, I don't see you as Black… your thinking is different.” Other times they were treated as interchangeable with other racialized people, for example, being asked to translate for clients who spoke completely different languages. This interchangeability is linked to tokenism, a kind of superficial inclusion that does little to nothing to change actual power dynamics. One therapist noted that after colleagues or clients found out her ethnicity, they would begin recounting previous experiences: “They’ll go into stories about other [Asian] people that they’ve met in their lives, and they’ll talk about, ‘Oh yeah, I had this one neighbour who was [Asian]. They were super nice.’”

Tokenism sometimes slides into exoticism (Essed, 1991), when the racialized person is positioned as an intriguing, even mysterious other. The gaze of curious observation may carry a message of not-belonging, as one participant suggested:

I usually eat lunch with my colleagues, right? And they’ll ask me what I’m eating. They’ll ask about the food. It's interesting, because everyone else is having lunch, but *my* lunch becomes “the” topic of conversation… They’ll ask what that is, and they’ll talk about experiences they’ve had at restaurants, where they’ve tried this certain food. They’ve enjoyed it or didn't enjoy it. So I feel like a lot of it comes out when we’re eating lunch, where, like, me being Asian, eating Asian food, becomes the centre of attention.

One therapist in an academic setting was chatting with students informally in a classroom when a student suggested she dance for them, a provocative form of exoticism given her North African heritage.

### Vicarious Racism

Lastly, interpersonal racism can be vicarious, directed against someone else yet still damaging. For example, hearing clients speak disparagingly about immigrants and refugees was challenging: “You get really tired of hearing that, over and over again.” Hearing colleagues describe therapists from other countries as backward, poorly educated and incompetent was hurtful. Witnessing clinicians refuse to accept racialized students for fieldwork placements, or listening to racialized students who struggle daily with racism in academic and fieldwork courses was painful. Racist assumptions about others could just as easily apply to the racialized therapist: “When my colleagues are imposing that on patients, what makes me exempt? Nothing, really, absolutely nothing makes me exempt.”

Vicarious racism can have ripple effects, as one participant described when an incident affected a client, a student and a preceptor:

A major incident happened in the past year, that I’m still quite angry with. I had a nursing colleague who was reviewing the file of a client, who was [Caribbean], and who was diagnosed with schizophrenia. She said that we shouldn't take him into the service, because he's [Caribbean] and they are slow and lazy. (sigh) Yeah. So, she said that in front of a student, who is of the same descent.

Vicarious racism is particularly difficult to respond to, as actions or comments directed at someone else nonetheless convey painful messages to witnesses about their own value. This preceptor ended up embroiled in a losing battle on behalf of the student.

## Institutional Racism

Institutional racism in the profession was noticeable as soon as participants entered occupational therapy education. They were frequently “the only one”: the first Black student, the only Middle Eastern student, and so on. Asian students in larger cities often found a small group of other Asian students in their programs, and typically stuck together. None of the Black therapists had ever worked with another Black therapist, though they had collectively over 50 years of experience.

In terms of hiring processes, several participants described submitting dozens of job applications and never knowing whether racism was the reason they did not get job interviews, though, “Classmates who hadn't had similar placement opportunities, later got the job and I didn't.” One participant was surprised to never get even an interview when she moved to a new province with over 20 years of experience. Another therapist thought she eventually got a hospital position nobody else wanted.

A few participants thought racism had hindered promotions for them, but this was difficult to prove. Nonetheless, institutionalized racism surfaced in the absence of racialized people in the higher echelons of workplace hierarchies. For virtually every participant, never seeing racialized faculty or health care leaders signalled the whiteness of the profession.

Elsewhere in the hospital, there's between 1/4 and 1/3 of the staff that is of a visible minority. But not in management. There is no person in leadership or management or a VP role, of a visible minority. None. … Not one manager. So, I’m pretty angry at that. I’m angry at the fact that they won't even acknowledge that that's a problem.

The absence of racialized leadership contributes to institutional nonresponse to racism. When clients refused to work with racialized therapists, managers rarely addressed the racism, they simply sent the therapist back or replaced them. Both responses demonstrate institutionalized racism.

Interpersonal and institutional racism intersect when flagrant racism from colleagues and clients is met with institutional denial and management inaction. In the incident above between a nurse and a student, the student followed up with a manager and the preceptor followed up with the Director, arguing for the creation of a diversity and inclusion committee—which was seen as “a great idea!”

Nothing came out of that. To my knowledge, there's no diversity and inclusion committee. I don't know what the manager said to the Director, but it sounds like it was along the lines of, “The nurse who made the comment is the real victim here, because the student made way too big of a deal of such a small innocent comment.” I don't know. So (sigh) I just kind of wanted to quit.

Framing the person experiencing racism as “the problem” ensures racism never gets taken up at the institutional level. At the same time, the need to always pursue action, and the resultant institutional inaction, can be exhausting. One participant experienced this vicariously in an academic setting, when students faced high levels of racism with little institutional recourse.

I had a student on Monday in my office, crying. She's racialized… her and a couple of her peers have put together a document outlining the racist behaviours of a clinical supervisor, that was poo-poo’ed as them being too sensitive.

Lastly, institutional racism is evident in the systematic ways racialized therapists were required to do extra work. One participant carefully crafted strategies to ensure client racism directed at her did not negatively affect other members of a therapy group. Some found working in ethnoracial minority communities meant client needs were often complicated by poverty, lack of insurance, need for advocacy and translation: “It's so much extra work for me… and because my case load was mostly like that, I ended up, it just made me feel burnt out a lot faster.” Also, when therapists shared their culture and language, while this could aid rapport-building, at the same time clients tended to raise a lot more issues and concerns, feeling relatively safe and understood. One participant said clients would raise with her all the issues they were unable to raise with their physiotherapists, their social workers and other professionals, due to language barriers.

## Structural Racism

Structural racism concerns broader sociocultural ideas that shape and justify inequitable access to social benefits. In participant narratives, this emerged in how professional authority and expertise of racialized occupational therapists were undermined, and how White Western epistemologies subjugated and invalidated other ways of knowing. Participants’ authority was undermined by appearing “too young,” using English that was “too accented,” and being mistaken for cleaners or other staff. Participants found their ideas were not taken seriously unless raised by others. Theories, frameworks, and assessment tools were described as relying on normative cultural expectations, pathologizing other cultural patterns and understandings, and failing to take any type of oppression into account.

I would say that any kind of discrimination at all, including the ones that can come from being a visible minority, is simply not taken into consideration with any of the evaluations, any of the interventions that we learn in school. As a little side project, I’ve actually been doing my own personal research, in terms of interventions for other populations, just because I feel like I don't have the, I wasn't given the proper tools and now I’m developing a few, but I feel like I still don't have enough.

Confronting the whiteness embedded in the profession takes extra, invisible work. One participant described “all the invisible work” required of racialized therapists—to create new resources as well as to navigate racism—as akin to getting an extra degree:

You need to learn and read and develop and relationship-build and all this stuff, so … you can understand and name your own experiences for yourself… depersonalize systemic racism, when you need to, name it when you need to. It's all this extra cognitive—other than the emotional load and the social—there's that extra, the invisible PhD, I call it.

## Psycho-Emotional Harms of Systemic Racism

The extra work demanded is just one of the harms inflicted by systemic racism in occupational therapy. Some participants described a kind of hyper vigilance, the need to be constantly watchful, aware, and guarded against casual injury. For example, one therapist became “A little bit more cautious” after negative feedback from students. After a client complained about her race, a participant remained guarded: “There was always the question in the back of my mind, every home that I walked into, ‘Will I be accepted? Will I—how will they react to me? Is this going to be an issue?’” In private practice, she debated whether to post her photo on her website, “Because it’d be visible that I was African Canadian. Would that draw in customers or would that turn people away? … The fact that I even had to have a conversation about that in my head…”

Undermining confidence is another result of racism. For example, one participant found her “confidence issues” and insecurities increased in university where she felt judged due to her race and working-class origins:

I see my classmates, and they come from a better background and language was never a barrier for them, and they’re Caucasian, and they would be able to speak without thinking and they would speak very fluently, and they were always very confident. And that made my confidence suffer a bit… Now that I’m a professional, it's the same thing. There's a lot of self doubt… I always feel like I’m not really fitting in.

Another therapist said when people doubted her competence, she began to doubt herself: “It kind of feeds into that.”

Participants described feeling frustrated, angry, and hurt when encountering racism, yet as professionals, they felt obliged to transcend those emotions in their work with clients and colleagues:

With clients that have been sort of rude to me… if that person didn't ask for another OT, and I continue to be their OT, providing services, of course inside I will be hurt from what they say, but I don't think I will treat them differently after that. I will still treat them the same way I treat any other client. I might be more cautious… a little more careful with how I say things.

Given typical management nonresponse, some participants had to work extra hard to “win over” clients who initially expressed overt racism towards them.

One participant had quit a job over racism, “because working for the institution is just too painful and just too frustrating, and I can't do that anymore.” Others had changed positions, and more had wanted to quit.

If I wasn't strong-minded, or maybe if I didn't have responsibility to look after my children, at a certain job probably I would have quit because all the bullying and all those things that were going on, I would have walked away.

One participant articulately described the ontological harms of relentless systemic racism, both direct and vicarious:

I’ll be honest; I’m going through a total, existential, “I don't want to be in the world” kind of moment. (laugh) Like, when someone says, “Hey, how you are doing?” I just want to say… “I’ve encountered four instances of systemic racism and it's only eleven o’clock in the morning. How do you think I’m doing?” … Really, really heavy… I am currently in a space of moral fatigue.

## Responses to Racism: Coping and Resisting

Participant responses to racism ranged from individual to collective. Some engaged in meditation, yoga, art, poetry, substance use, or reading: “I try to do self-care and to have a meditation practice and do more yoga, because otherwise, I am going to be the stereotypical angry Black woman. (laugh) And I sometimes am regardless.” Coping, surviving, is often all that is possible; sometimes people find the energy to resist, collectively and/or individually—and there are usually costs, particularly in terms of stereotyping and scapegoating.

### Making Sense of Racism

Finding ways to make sense of racism is one way of coping, a survival strategy—though arguably it could also be seen as a profound ontological harm inflicted through racism. Many participants expressed doubts, questioning whether specific struggles were due to racism or not. Some displayed self-blame, taking responsibility for negative treatment.

I should just tell myself not to think of that [my class and race] so much… I almost make it into a barrier, because I make the assumption that people are judging me because of my background, when it could actually be myself. And then I feel like I’m creating my own barriers, by using that to rationalize why situations don't go well, or why this is happening to me… But that may not be the actual situation, it could be just the perception I have.

Some participants absolved others from blame, for example, suggesting that if you just respond the right way, you can end the racist comments and innuendos. Some suggested racism signalled ignorance: “I am not blaming them… they lack the awareness.” 

Minimizing or downplaying racism, some participants thought it could be due to other things, such as mental illness, or past negative treatment, or personal dislike—a kind of “generic harm” perspective.

No matter what your background is or where you come from, in the profession we are always going to run into clients that might be going through a lot. And I don't know if it's because of that or it might be the nature of who they are, they might pick on things to attack, to attack you.

Some pointed out that they deal with racism in every facet of their lives, and it is no better nor worse in the profession. Relativizing was also common, suggesting things could be far worse elsewhere. This came up mainly in suggestions that people in smaller cities and towns are more racist, more “narrow minded” and “not exposed to different cultures.”

Probably the most common way of making sense of racism was to dismiss it, find a way to take it in stride. Most participants at least occasionally chose to ignore racist incidents: “I just sort of let them roll off my back.” As one therapist said, it “hurts in the moment, but … I try not to take it personally (laugh).”

### Choose Your Battles: Avoidance, Withdrawal, and Engagement

Participants repeatedly stated it is essential to “choose your battles”: decide when to resist racism, and when that is not worth the cost. Sometimes therapists simply exit or shut down discussion. For example, after a challenging interaction with a client, one person decided, “there's a point where I have to be self aware and excuse myself from a situation that might be detrimental to me.” Politely disrupting a racist discourse and leaving is an acquired skill. In another form of disengagement, some participants withdrew from all parts of their jobs except work-related tasks: “Work is work. Clock in, clock out, do a good job and then leave.” 

Choosing to engage, to actively resist racism, meant assessing costs and benefits. Some participants were less likely to challenge racism when it came from clients than from colleagues; they had to find careful ways to approach clients, whereas staff “should have known better.” Some linked resistance to increasing job security: “I don't care anymore. And I can say that because I have job security… I have more resources that I can rely on. I don't necessarily think I’m safe, but at this point, it's like, ‘Well, Hell be damned.’ (laugh).” Participants were clear that resistance entailed risks:

It's so important for me now, to be more vocal about it. 'Cause if I’m not vocal about it, then I’m actually allowing this to continue, right? So this is why I’m putting myself out there, and being more vocal, and at the risk of making myself look like a trouble maker. 'Cause that's what I’m going to look like. I’m going to look like the trouble maker, right? And that's how I’ll be scapegoated. So, it's either I say something about it, and risk that or say nothing, right? And at this point in my life, I’m really geared towards, “It's time to say something.”

Another participant strived to weigh the perception that she raised racism “too much” against the potential value of speaking: “I do try to talk about it at a judicious time, I have to pick and choose. Unfortunately, *any* kind of comment that's made about race is considered stirring the pot.”

### Community, Collective Responses, and Suggestions for Change

At the collective level, finding or forging the community was endorsed by all participants. Some joined networks of racialized therapists or found one or two other racialized health professionals to decompress with. Sharing experiences, interpretations, strategies, “no matter what it looks like—community. You need your people. You need to find spaces where … you’re not modulating your performance; where you’re just you and who you are is not just good enough, it's awesome.” Forging community breaks the isolation and strengthens resistance.

Participants also argued for systemic change, such as educating racialized youth to envision entering occupational therapy, providing stable funding for racialized students, as well as supports for navigating application processes, and for language differences. Schools must make space for different ways of learning. Seeing racialized faculty in occupational therapy schools might help disperse the monolithic whiteness of the profession. Participants suggested organized peer support groups among racialized students, along with a mentoring network connecting racialized students and therapists, could simultaneously provide peer support among therapists, potentially encouraging leadership roles.

One therapist spoke of embracing a focus on the future, seeing her work as being so that “those that come next can see the traces of the work already done.” Yet, strategies for change within the profession were scant. One therapist worked with colleagues to enhance cultural competence among all health professionals, concerning racism and other forms of oppression. One person challenged human resources to better implement employment equity hiring processes. Another worked with human resources and managers to reduce disadvantages to racialized applicants in hiring processes.

## Discussion

Participants experienced racism at interpersonal, institutional, and structural levels, all intricately connected (Nazroo et al., 2019). While this is undoubtedly not surprising for racialized readers, it is important to note how the occupations of racialized therapists are hindered by racism within a profession that claims to confront inequity. White supremacy may seem insurmountable, too complex to tackle, but it is also part of everyday interactions, practices, and policies. These “closer-to-hand” instantiations of systemic power structures may be more immediately amenable to change.

Instances of interpersonal racism were perhaps most obvious. Exclusion from social events, direct or indirect derogatory comments by colleagues and clients, irate clients, rejection of your accent or your actual services as a therapist—these were painful experiences causing psycho-emotional harm. Even when framed positively or admiringly, tokenism and exoticism (Essed, 1991) can feel like being pinned to a specimen board for curious observation; the message of not-belonging is barely hidden. Navigating interpersonal racism is exhausting and stressful.

Yet in the context of occupational therapy, interpersonal racism is compounded through an intersection with institutional and structural racism. When individual experiences of racism receive no meaningful response from managers, the institution perpetuates racism. When there are no racialized managers that both embody and exacerbate institutional racism. When the assessments, theories, models and frameworks taught, learned and practiced in occupational therapy encompass only white, Western worldviews, this is an institutional sedimentation of structural racism (white supremacy, [Grenier, 2020]). Basic concepts and core professional values such as “client-centeredness,” which purport to be race-neutral, are revealed to embody whiteness when systemic racism is taken into consideration. What does client-centeredness look like for a racialized therapist when a client is overtly racist to them? When managers and institutions cannot (or choose not to) see the racism? When processes are designed to support the (presumed powerless) client, denying the coexisting power structure of systemic racism? For racialized therapists, their presumed “power over” in relation to clients is always complicated by racism (Beagan et al., forthcoming).

### Making Sense of Racism

The multilayered harms occasioned by systemic racism demand that racialized occupational therapists find ways to cope, survive, and potentially resist. Beyond individual self-care, participants needed to engage in cognitive sense-making strategies to find a place for racism. As others have found, these strategies include downplaying, minimizing, ignoring, self-blaming, justifying, individualizing, and relativizing (Kristoffersson et al., 2021; Perkins et al., 2019; Vazir et al., 2019). This sense-making is a survival strategy, a way of getting on with everyday life. Particularly when racism is subtle, and so thoroughly ingrained in social norms, people may be able to identify incidents of racism yet hesitate to name them as such, “point[ing] to a deep ambivalence and mistrust in their own experiences of reality. … This may be one of the most insidious and exhausting things about being systematically disadvantaged: to be constantly guessing and doubting your experiences” (Kristoffersson et al., 2021, p. 7). Such ambivalence and self-doubt (Cottingham et al., 2018) are part of the ontological violence (Goodley & Runswick-Cole, 2011) of racism.

In their study of the race-talk of Black Americans, Perkins et al. (2019) found the discourses available for sense-making all had negative implications. Their participants employed discourses that explained away, downplayed or took responsibility for racism. Though these strategies may perpetuate racism, rendering it less visible, they are nonetheless important means of coping. If “everyone is discriminated against for something,” I need not come to terms with myself as a specific target. If racism is a matter of “ignorance” or age or politics, I can hope change will come. If racism is the result of my own negative behaviours, then if I do all the “right” things, I may evade racism. If I still experience racism, I must be at least partly to blame. Very similar sense-making strategies were found by Kristoffersson et al. (2021) among medical students: “To relieve the pain of oppression it was transformed into something less threatening. Further, by comparing their experiences with racism elsewhere, a picture was maintained whereby their medical school was, if not free from racism, then at least better than other contexts” (p. 7). Similarly, Vazir et al. (2019) found Canadian physiotherapists downplayed, doubted, and ignored racism, which, of course, does not mean racism is not operative. In those studies, as in this one, racism structured everyday work within the profession.

While the harms of racism in occupational therapy are borne by racialized therapists, the responsibility for change need not—must not—lie solely with them. If the profession is built on the foundations of white supremacy (Grenier, 2020), if whiteness is written on the bones of the profession, widespread commitment and action are needed to effect change. Participants have identified some strategies for change, such as peer supports, mentoring and community building among racialized therapists and students. Although very helpful for individual and small groups of racialized therapists as a mechanism to survive, such initiatives do little to address the institutional and structural forms of racism that pervade the profession (see Emery-Whittington, 2021). Additionally, Nixon (2019) has stressed the importance of “practicing critical allyship,” wherein members of dominant social groups tackle the work of dismantling oppressive systems of power. It is the responsibility of white people (too) to interrogate the ways whiteness is built into business-as-usual in occupational therapy. This starts with learning more about racism and white supremacy, listening to the guidance of racialized people, acknowledging failures to value and respect the expertise and authority of racialized people and recognizing that expertise, and beginning the work of transforming institutions. It means recognizing and challenging white fragility, the fear/anger/guilt/defensiveness that leads white people to go silent, debate, argue, withdraw, or resist when white privilege is surfaced (DiAngelo, 2011).

In medicine, Kristoffersson et al. (2021) have suggested the value of bystander training, helping white people to identify and respond to racism, disrupting the normative passive complicity with racism. Minimally, occupational therapy education must incorporate structural analysis of racism, helping future therapists understand how it is infused throughout society—and the profession—and how it may be undermined. This means moving from cultural competency education to antiracist education (Cottingham et al., 2018), both in curricula and in accreditation standards. This is a needed first step to understand the entrenchment of systemic and institutional racism, but insufficient to dismantle the structures that perpetuate it. The need to move more racialized therapists into leadership positions and faculty positions is urgent, essential to begin disrupting pervasive institutional racism (Iheduru-Anderson & Wahi, 2021), rather than relying on the next generation of therapists to bring about change.

As Ramugondo (2018) suggests, deconstructing colonialism within occupational therapy requires attending to hierarchies of power, respecting differing knowledge (and knowers), and recognizing the full humanness of those who have been rendered inferior, those who are not white and from the global North. The suggestions for change put forward by racialized and Indigenous therapists are essential starting places for changes urgently needed, demanding critical interrogation, if not dismantling, of the theories, systems, models, frameworks, institutions, and practices that structure the profession (Emery-Whittington, 2021; Gibson, 2020). Highlighting the harms done to racialize therapists through racism within the profession is only of value if it is a step towards systemic change. The harms documented here are part of “business-as-usual” in occupational therapy in Canada; confronting racism, undermining white supremacy perpetuated through practices and assumptions, small and large, must become the new normal.

## Conclusion

Systemic racism operates through interconnected social patterns of power relations that privilege some and hinder others on the basis of racialization. This qualitative study suggests racialized occupational therapists in Canada experience racism at all levels (interpersonal, institutional, and structural) hindering their productive occupations. Those levels connect in mutually reinforcing intersections, causing ongoing harm to racialized therapists. Though they employ numerous coping strategies, including sense-making and sometimes resisting, the costs are high. The profession, and particularly white people in occupational therapy, have an obligation to develop the skills to recognize and analyse racism at multiple levels, and to enact critical allyship (Nixon, 2019), working with racialized therapists to dismantle this system of power. Meanwhile, building community supports for racialized therapists and students within the profession, and ensuring racialized therapists advance into leadership positions are critical steps.

## Key Messages

- Racism is normalized in the profession; racialized occupational therapists experience it at interpersonal, institutional, and structural levels, causing extensive psycho-emotional harm.
- Along with self-care and avoidance, therapists engage in resistance and collective responses. Trying to make sense of racism is one pervasive coping strategy.
- White people in occupational therapy have an obligation to develop the skills to recognize and analyse racism at multiple levels, ultimately acting with racialized therapists to dismantle this system of power.
